# Recent Insights into Glucose-Responsive Concanavalin A-Based Smart Hydrogels for Controlled Insulin Delivery †

**DOI:** 10.3390/gels10040260

**Published:** 2024-04-11

**Authors:** Maria Bercea, Alexandra Lupu

**Affiliations:** “Petru Poni” Institute of Macromolecular Chemistry, 41-A Grigore Ghica Voda Alley, 700487 Iasi, Romania

**Keywords:** concanavalin A, glucose-responsive system, polysaccharide, hydrogel, biosensor, controlled insulin delivery, diabetes theranostics

## Abstract

Many efforts are continuously undertaken to develop glucose-sensitive biomaterials able of controlling glucose levels in the body and self-regulating insulin delivery. Hydrogels that swell or shrink as a function of the environmental free glucose content are suitable systems for monitoring blood glucose, delivering insulin doses adapted to the glucose concentration. In this context, the development of sensors based on reversible binding to glucose molecules represents a continuous challenge. Concanavalin A (Con A) is a bioactive protein isolated from sword bean plants (*Canavalia ensiformis*) and contains four sugar-binding sites. The high affinity for reversibly and specifically binding glucose and mannose makes Con A as a suitable natural receptor for the development of smart glucose-responsive materials. During the last few years, Con A was used to develop smart materials, such as hydrogels, microgels, nanoparticles and films, for producing glucose biosensors or drug delivery devices. This review is focused on Con A-based materials suitable in the diagnosis and therapeutics of diabetes. A brief outlook on glucose-derived theranostics of cancer is also presented.

## 1. Introduction

Abnormal glucose metabolism is a metabolic disorder that causes diabetes, a disease that requires the close monitoring of blood glucose and clinical treatment by the daily adjustment of insulin doses while also making an appropriate dietary choice. In such cases, the long-term stabilization of the normal plasma glucose level becomes difficult to achieve, and various unwanted complications can appear [1,2,3]. Many efforts have been undertaken to improve diabetes management, mainly focused on continuous glucose monitoring and developing smart insulin delivery systems. Thus, new multifunctional theranostic systems are designed using glucose-sensitive materials that can indicate the changes in blood glucose levels, ensuring the real-time delivery of insulin [4,5,6,7].

In the last few decades, the following categories of glucose-responsive systems have been developed [2,8,9,10,11]:

(1) *Environmental changes catalyzed by enzymes*. The method involves the use of glucose oxidase (GOx) as a catalyst enzyme for the transformation of glucose into gluconic acid [12,13,14,15]. The reaction leads to a decrease in the environmental pH, the consumption of O_2_ (hypoxia), and the formation of H_2_O_2_. GOx was formerly used for insulin delivery systems, but it has some limitations in practical applications, for example, a response lag for insulin release [16,17]. This method has been recently used to trigger glucose-sensitive microneedle systems (MNs) [10].

(2) *Using the glucose-binding molecules.* The reversible conformational change in glucose binding proteins (GBPs), induced by the presence of glucose, determines a quantitative “accordion”-like dynamic response [18,19,20,21,22,23]. Among different GBPs, concanavalin A (Con A) is one of the most common lectins suitable for achieving glucose-triggered insulin delivery. Con A causes a gelation of polysaccharide or polymers containing glucose moieties, forming a glucose-responsive network [6,8,23,24,25,26,27,28,29]. The basic principle of action of these hydrogels is the competitive non-covalent binding of glucose and glucose derivatives to the four binding sites of Con A. The insulin is displaced from Con A by glucose in response to a corresponding amount of glucose present in the environment. The migration of insulin in the sol phase is faster than in the gel phase, and thus the insulin release is controlled by the glucose concentration in the environment and the diffusion phenomenon [6].

(3) *Molecular recognition by chemical diol binding moieties*. Polymers with phenylboronic acid (PBA) moieties are able to form complexes with hydroxyl groups of cis-1,2 or cis-1,3 diols in glucose and other similar diols, acting as molecular receptors for saccharides. The presence of glucose determines borate crosslinking, and the formed hydrogel is able to release more drug as the glucose concentration increases [30,31,32]. The boronic acids–saccharides’ affinity is not very high. However, as a result of binding glucose, PBA undergoes a change in optical properties that can be detected by various methods, such as fluorescence or surface plasmon resonance [11]. Typically, the physiological glucose concentration varies from 2.5 mM to 50 mM [11,33].

(4) *Unconventional treatments* were also explored in order to avoid complications caused by hyperglycemia episodes. In the absence of a pancreas transplant, the “closed-loop” stimuli-responsive systems [34], which can mimic continuously and automatically the activity of the pancreas, were tested. A first strategy was the use of a glucose-responsive matrix able to give a suitable response to losses or gains of glucose levels. A second approach involved the chemical modification of insulin by introducing functional groups sensitive to glucose [35]. Glucose-responsive drug delivery is one of the most prevailing methods of monitoring blood glucose levels (BGLs) and releasing insulin when an increase in BGLs is detected. Among other “closed-loop” systems, glucose-responsive MNs are endogenous drug delivery devices that are more easily accepted for long term use as compared with traditional injections, being characterized by simplicity and adaptability. The “artificial pancreas” systems can work according to different principles presented above, i.e., Gox, Con A or PBA electronic sensor-based MNs, which detect elevated BGLs and supply insulin. A reversible swelling of the matrix occurs as a response to hyperglycemia, delivering insulin into the blood flow [5,36,37,38].

(5) *The use of glucose-binding apoenzymes* [11]. As an example, apoenzyme (Apo-GOx) is a reversible non-consuming sensor for glucose, obtained from GOx by removing the coenzyme. Such enzymes act as glucose-binding (not metabolizing) molecules, similar to Con A, when they undergo a decrease of the intrinsic tryptophan fluorescence (up to 25%) [39]. The fluorescent labeling of proteins [40,41] offers more opportunities; the choice of a proper label allows for a fine tuning of the optical properties. The glucose concentration range covered by the proteins is broad, and in the case of blood glucose, genetic engineering has further moved the dynamic ranges towards higher concentrations.

Blood glucose monitoring is important for the careful management of diabetes, a disease that affects a large part of the population. The number of adult diabetic cases increased from 108 million in 1980 to 422 million in 2014. The International Diabetes Federation estimated the percentage of adult diabetic cases in 2021 as 10.5% of the global population (about 537 million), and it is expected to escalate to approximately a billion by 2045 [42,43,44].

BGL values within the normal limits mean concentrations between 70 mg/dL (3.9 mmol/L) and 100 mg/dL (5.6 mmol/L) [45], which avoid a series of drastic complications (limb amputation, kidney failure, heart disease, etc. [1,2]). Normally, the fasting blood glucose (FBG) concentration is below 5.6 mmol/L. FGB could be considered an independent predictor of an adverse 90-day outcome in patients with acute ischemic stroke. When FBG ≥ 5.5 mmol/L, the risk of a 90-day unfavorable outcome significantly increases [46]. Understandably, diabetes and glucose sensing materials are major public health concerns.

Among the different alternatives, concanavalin A appears to be a suitable selective and biocompatible natural protein, easily in vivo administered by using smart systems with appropriate pharmacokinetic behavior [6,8,24,28,29]. After a gap of about 20 years, when only a few studies were reported, the interest in studying Con A seems to be growing again, as it is reflected by the increasing number of publications in the last 15 years.

The main aspects presented in the literature will be briefly discussed in the following sections, with an emphasis on the new safe and bioinspired hydrogels suitable for glucose detection and insulin delivery systems, capable of maintaining the blood glucose level within the normal limits. The incorporation of Con A into hydrogel-based materials can be considered a minimally invasive method to achieve glucose sensing and for further integration into a smart glucose biosensor. Developing a glucose biosensor is a complex work that requires multidisciplinary skills.

## 2. Origin, Structure and Functions of Concanavalin A

The specific interactions of glucose moieties with binding lectins were investigated for decades to understand the functions and mechanism of lectins’ association with specific ligands [47].

Mannose-binding lectins, isolated from plants, algae or fungi, present different structural scaffold structures which contain mainly carbohydrate-binding sites with high specificity, able to recognize the glycans containing mannose and to reversibly bind them [48]. Among plant lectins, and particularly legume lectins, concanavalin A (Con A) and Canavalia brasiliensis (Con Br) were deeply studied for several decades. There is a strong relationship between the structure and biological activity of these two lectins; thus, small shifts in the amino acid sequences change the tertiary and quaternary structures with high consequences in their biological activity [23,47,49].

Con A, firstly isolated over one century [23], exists as a dimer in solution [50]. Each monomer presents one saccharide-binding site and two metal-binding sites for a calcium ion (Ca^2+^) and a transition metal ion (Mn^2+^). The structural aspects were investigated by X-ray and infrared spectroscopy, evidencing 237 amino acid residues [51,52,53,54].

As sources for Con A, either the isolation from Jack beans (*Canavalia ensiformis*) [23,55] or biosynthesis [56] can be considered. The purification of Con A involves firstly the cleaning of the seeds followed by grinding them to a fine powder. Then, the extraction of soluble proteins is conducted using buffer or saline solutions, in the presence/absence of divalent salts (CaCl_2_ and MnCl_2_). The extract is fractionated either by precipitation with ammonium sulfate or by chromatography with an immobilized mannose or dextran matrix (Sephadex G-50) [55]. Con A of high purity can be obtained by applying either gel filtration, ion-exchange chromatography [49,57], crystallization in different conditions [23], a glucosylated magnetic nanomatrix [55] or using a two-phase system composed of 22% (*w*/*w*) poly(ethylene glycol) (PEG) of 8 kg/mol and 12% (*w*/*w*) citrate, at pH = 6.0 [58].

**Figure 1:**
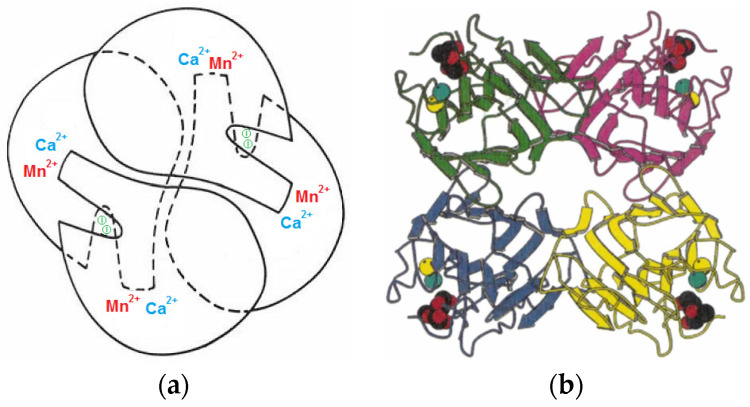
(**a**) A schematic presentation of the Con A tetramer activated by the presence of Mn^2+^ and Ca^2+^ ions. Adapted with permission from [52], copyright 1975, Elsevier. (**b**) A ribbon representation of the Con A tetramer: Mn^2+^—yellow circles; Ca^2+^—cyan circles; the two dimers formed are shown by green-pink and blue-yellow subunits. Adapted with permission from [50], copyright 2007, Wiley.

The most important feature of Con A is the high affinity for reversibly and specifically binding glucose and mannose [47]. It is a hemagglutinin, constituted by subunits containing two large antiparallel pleated sheets, which form dimers and tetramers via interactions of the pleated sheets, folded in dome-like structures of 42 × 40 × 39 Å related by 222 symmetry forming tetrahedral tetramers (Figure 1) [51,52,59]. Each of the four identical subunits has the molecular weight of 2.55 × 10^4^ g/mol [60]. The protein has an The isoelectric point of protein, pH_i_, is about 5.1 [61]. The solubility of Con A in water is influenced by temperature, pH and ionic strength; the reported values range between 1 mg/mL and 50 mg/mL [62]. Furthermore, the supramolecular structure of protein is very sensitive to the environmental pH. Thus, below pH = 6, the Con A subunits associate and form predominantly dimers; at about pH = 7, the tetramers’ formation prevails [63,64].

The lectin isolated from Japanese red sword beans (*Canavalia gladiata*) showed specificity to glucose, maltose, mannose, methyl-D-mannoside and thyroglobulin and it was not sensitive to rhamnose. This lectin can be used to prepare smart hydrogels for insulin’s controlled release [24,65,66] or as a bioactive protein in medical research, for example, as a potential agent for cancer prevention [67,68].

For the optimal binding of one saccharide molecule, each monomer unit of Con A with 237 amino acid residues must include one Ca^2+^ ion and one Mn^2+^ ion. When the two divalent cations are removed from the protein in acidic medium, Con A is no longer able to bind saccharide molecules. The saccharide binding activity is restored in the presence of the Ca^2+^ and Mn^2+^ cations, under specific pH conditions, namely pH = 7.4 [24,65,66]. An inhibitor of Con A activity, β-(o-iodophenyl)-D-glucopyranoside (β-IPG), was also identified in the protein structure (denoted I in Figure 1a).

Each cation is surrounded by six ligands (four from the protein and two from water molecules) which form binuclear complexes of two octahedral coordination shells sharing a common edge. In the case of a Mn^2+^ ion, one molecule of water is connected through hydrogen bonds to the cation and protein residue; the second one is located in the shallow channel exposed to the surface. The protein ligands are connected through the acidic part near the amino-terminus sequence. Mn^2+^ ions bring the amino-terminal parts of the chain into appropriate conformations that create part of the Ca^2+^ binding sites on the opposite end of the chain [69]. In the case of a Ca^2+^ cation, the two water molecules form hydrogen bonds with the neighboring groups of the carboxyl-terminus. Structural and thermodynamic analysis has shown that water molecules mediate the weak ligand–protein interaction by anchoring the mannoside ligands to Con A, whereas the binding free energy is enthalpy-driven [70]. The Con A tetramer is able to bind reversibly the α-anomer of D-mannose of various glycoproteins or glycolipid receptors on the cell surface; specifically, it binds to nonreducing terminal α-D-mannopyranosyl, α-D-glucopyranosyl or N-acetyl-D-glucosamine and to unmodified C-3, C-4 or C-6 hydroxyl groups of polysaccharides. This lectin also binds to the lymphocyte surface, stimulating a large percentage of lymphocytes [52,71,72]. α-Mannosyl residues substituted at their C-2 position also bind to Con A [73]. The high affinity of Con A was observed for the branched trisaccharide Man(α1-3)[Man(α l-6)]Man, as occurs in other N-asparagine-linked glycoproteins [74,75,76]. Similar behavior was reported for branched mannose oligosaccharides and glycopeptides, occurring via the Man(α 1-6)[Man(α 1-3)]Man trimannosyl moiety of the α-1,6-antenna of the carbohydrate chains [77]. Non-polar binding sites present on the Con A molecule interact with phenyl β-glycosides of mannose, glucose and N-acetylglucosamine. Also, there are low affinity sites, located per each subunit of Con A, that bind tryptophan, indole-3-acetic acid and non-polar molecules [72,78].

Lectins are considered potential alternatives to produce plant-based products for treating infections. They interact with carbohydrates from the bacterial cell wall or fungal membrane and damage their structure [79,80]. Due to the strong specificity in the presence of glucose, the Con A networks appear promising for clinical use [81]. In addition, some studies suggest that lectins are potent antiviral agents against coronavirus, i.e., inhibitory activity against SARS-CoV-2 [82].

The competitive binding of glucose to Con A-based materials presents a high applicative potential for improving glucose monitoring devices. Some aspects discussed in the literature are briefly presented in the next sections.

## 3. Con A-Based Glucose-Responsive Materials

For several decades, many efforts were undertaken to develop physiologically relevant biomaterials. In this context, glucose responsive systems able to control BGLs or self-regulate insulin delivery are of high interest.

Insulin is a peptide hormone composed of 51 amino acids, secreted by the pancreatic β cells, that plays a significant role in the modulation of a wide range of physiological processes, cell growth and glucose homeostasis [83]. Insulin restores normal blood glucose levels and normalizes the storage of glucose in the muscles, adipose tissues and liver. Abnormal levels of insulin in the body have been linked to several chronic diseases [84], and the “insulin era” in treating diabetes represented huge progress [85]. Then, glucose-sensitive insulin delivery systems that can mimic β-cell function were developed to control BGLs and to avoid injection. The main concern of researchers is now to produce minimally invasive devices that real-time monitor the glucose levels and continuously release the required amount of insulin [4,36,86,87,88,89].

Con A presents a high affinity for glucose, greater than for glycosylated polysaccharides or glycopolymers [90], forming glucose-sensitive hydrogels. Among the most important parameters that play an important role in determining the strength of the saccharide–Con A complex, the enthalpy, entropy and steric stabilization of the complex must be considered. In addition, glycopolymers with stiff helical structures demonstrated effective attachment to lectin molecules [79]. Thus, the binding affinity of glycogen and mannan to Con A was evaluated by using quartz crystal microbalance [91]. The equilibrium association (K_a_) and dissociation (K_d_) constants were determined:

- for Con A–glycogen: K_A_ = 3.93 ± 0.7 × 10^6^ M^−1^; K_D_ = 0.25 μM ± 0.06 μM
- for Con A–mannan: K_A_ = 3.46 ± 0.22 × 10^5^ M^−1^; K_D_ = 2.89 μM ± 0.20 μM

Thus, Con A presents a higher affinity to glycogen as compared to mannan.

The thermodynamics of binding lectins with carbohydrates were deeply investigated by Mandal et al. [71,92] by using isothermal titration calorimetry. For Con A–core trimannoside, the following values were reported: K_a_ = 4.9 × 10^5^ M^−1^ and the heat of binding, H = -14.4 kcal/mol. The trisaccharide moiety 3,6-di-O-(α-D-mannopyranosyl) -α-D-mannopyranoside, which is present in asparagine-linked carbohydrates, binds Con A with a higher enthalpy change and 60-fold greater affinity than for the monosaccharide. Thus, the trimannosyl moiety was considered the primary binding epitope for Con A. It was also shown that Con A presents an extended binding site with high affinity for 3-, 4- and 6-hydroxyl groups of the (1,6)Man residue of the trimannoside, a site with lower affinity that bound the 3-hydroxyl of the (1,3)Man residue, and a third site that involved the “core” Man residue [71,92].

Con A presents therapeutic potential, being able to bind receptors on cancerous cells, such as MT1-MMP, activating immune cells to kill tumor cells [93]. It was shown that the addition of Con A to a mixture of specific activated cytotoxic T lymphocytes induces non-specific cytotoxicity by blocking the antigen-binding receptors of T cells [94]. However, as the authors underlined, an important number of receptors remains unoccupied, and Con A bonding does not completely suppress the cytotoxicity. Other in vitro and in vivo studies on Con A toxicity evaluated the health risks of an implantable biosensor (subcutaneous skin tissue). The small amount of Con A required for implantable glucose-sensitive detector devices (<10 μg/μL) does not harm health even if an unexpected damage of the sensor occurs [33]. Thus, Con A hydrogels are minimally invasive sensors, and they can be considered safe.

Glucose-sensing systems were designed by the non-covalent crosslinking of glucose-containing polymers and Con A that undergo a reversible gel-to-sol phase transition in the presence of free glucose. The phase transition occurs if the concentration of the free glucose in the environment is more than four times that of polymer-bound glucose [95]. The leakage of Con A from the hydrogels [21] represents one of the most important inconveniences. Therefore, Con A was immobilized as a glucose-responsive unit in a smart insulin delivery network by covalent bonds, avoiding its leakage into the human body [24,65,66,96,97,98,99].

Thus, based on the high affinity of Con A for glucose and mannose, different glucose-responsive polymeric materials, such as hydrogels, microgels, nanoparticles and films, were developed and investigated for producing biosensors or drug delivery devices [7,25,65,81,100]. In these formulations, Con A was used either in solution, trapped or recirculating within selectively permeable hollow fibers, either as complexes or crosslinked systems. The free glucose determines competitive binding on Con A sites leading to the dissociation of the Con A/polymer complexes. Insulin release occurs via glucose-stimulated swelling/contraction, dissolution, pore size change, charge reversal or polymer degradation.

In the following sections, the glucose-responsive insulin delivery systems based on Con A are briefly presented.

### 3.1. Hydrogels and Microgels Sensitive to Glucose

Generally, hydrogels consist of polymeric networks formed by chemically/physically crosslinked macromolecular chains that can swell but not dissolve in water. Different Con A-based hydrogels were designed, and they exhibit swelling changes in response to glucose concentration, presenting a high potential to be used as glucose biosensors and intelligent insulin delivery carriers. The response rate to glucose stimulus is influenced mainly by the specific interactions of glucose with the corresponding receptor sites of Con A that determine structural changes, influencing the swelling ability of the network in well-defined environmental conditions.

At physiological pH, the Con A tetramer containing four sugar-binding sites (Figure 1) acts as a crosslinker and reversibly interacts with pendant saccharide moieties of macromolecules. If the environment contains glucose molecules, the saccharide moieties are replaced by free glucose; the crosslinking density of the network decreases, and the swelling degree increases. Thus, based on the specific interaction between the polymer-bound glucose and Con A, glucose-sensitive hydrogels were prepared, showing dynamic reversible changes of the crosslinking points in response to the variation in free glucose concentration. The main application of glucose-sensitive hydrogels is in the development of self-regulating insulin delivery systems.

The self-regulated delivery devices contain a closed-loop insulin release system created for substituting pancreas activity. It contains a glucose-sensing component and a sensor-triggered insulin release part [35,101]. The release rate of insulin is self-adjusted in response to glucose levels in the blood. For such systems, the glucose sensitivity and cytotoxicity were tested and analyzed [21].

Polysaccharides [102] and synthetic polymers containing saccharide residues [103] may be complexed with Con A forming glucose-sensitive hydrogels. The involved mechanism is the displacement of the polymer chains from the lectin receptors by the incoming glucose molecules. As a consequence, the viscosity of networks decreases. The overall viscosity increases again when glucose is replaced by the macromolecular chains. This reversible process turns drug diffusion *on* and *off*, controlling drug delivery [102]. A less invasive treatment of the disease seems to be the use of a tissue-engineered pancreatic substitute consisting of non-beta cells and a glucose-responsive material based on Con A–glycogen sandwiched between two polycarbonate membranes. This hybrid material exhibited glucose-dependent sol–gel transformations and provided a fast physiologic regulation of insulin release in response to glucose concentration changes [104,105].

Some relevant research on polymeric hydrogels containing Con A is highlighted below.

#### 3.1.1. Smart Networks of Synthetic Polymers and Con A

A thermal denaturation of Con A occurs in a physiological environment (37 °C, pH = 7.4, 0.15 M NaCl). In addition, the electrostatic interactions with charged molecules or surfaces limit the efficacy and lifetime of competitive glucose binding. In order to improve Con A‘s stability in physiological conditions, glucose-sensitive hydrogels were prepared using PEGylated Con A, i.e., poly(ethylene glycol) (PEG) chains were covalently bound to lysine groups of the Con A surface [106]. PEGylated Con A hydrogels avoid the thermal instability of protein and its non-specific electrostatic binding and allow for in vivo continuous glucose monitoring and long-term controlled insulin release.

Allyl glucose was conjugated into polymeric structures by copolymerization with comonomers such as acrylamide, 3-sulfopropylacrylate or N-vinyl pyrrolidone [6,97]. Other membranes were constructed from crosslinked dextran (Dex) to which Con A was coupled via a spacer arm [97].

In other approaches, complexes of glycosylated insulin and Con A were prepared [107,108,109] and further incorporated into a polymer membrane pouch [110]. Macroencapsulated islets in highly substituted polysulfones—hydroxy methylated (CH_2_OH) derivatives with a high degree of substitution (1.7–1.8)—showed a rapid response to glucose stimulus with the kinetics and efficiency of insulin release similar to that observed of free-floating islets (control, without biomaterial) [111]. Such materials were proposed for the development of the bioartificial pancreas [112]. Materials containing succinyl-amidophenyl-glucopyranoside insulin and Con A enclosed in hydrophilic nylon microcapsules were also designed [113]. Both membranes and microparticles can operate for in vivo self-regulating insulin delivery [35,113].

A glucose-sensitive sensor was obtained by introducing Con A into the network of a synthetic polymer having pendant glucose, i.e., poly(2-glucosyloxyethyl methacrylate) [poly(GEMA)] [103,114]. The crosslinking density increases due to the supplementary Con A binding points, determining a decrease in the swelling degree and an increase in the turbidity. The presence of an increasing amount of glucose or mannose determines their selective binding to Con A sites and, at the same time, a dissociation of the poly(GEMA)/Con A complex. This is accompanied by the decrease in turbidity and an increase in the swelling degree, effects that are more pronounced in the presence of mannose than of glucose. In addition, the hydrogel swelling behavior remains unchanged in the presence of galactose. The solution becomes clear and the poly(GEMA)/Con A complex dissociates in the presence of glucose or mannose which form complexes with Con A.

**Figure 2:**
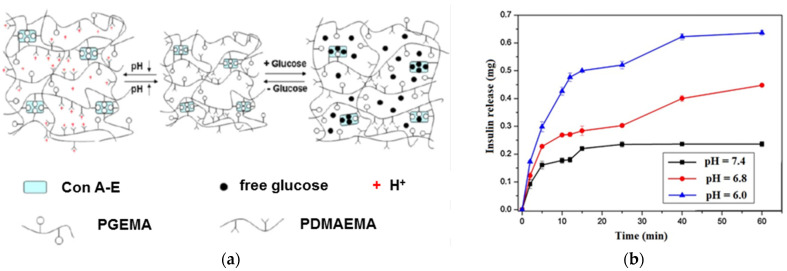
(**a**) Schematic presentation of structural changes in smart Con A-E/PGEMA/PDMAEMA microgel in response to glucose and pH changes; (**b**) insulin release during 60 min in condition of small pH changes from 6 to 7.4. Adapted with permission from [29], copyright 2011, Elsevier.

Microgels which present a fast response to glucose concentration in the environment when the pH value changes were designed for self-regulated insulin delivery, but they can also serve as actuators sensitive to glucose. Thus, glucose and pH dual-responsive networks with an average size of 38 μm for insulin release were prepared by the copolymerization of methacrylated concanavalin A (Con A-E)/glucosyloxyethyl methacrylate (GEMA) and N-(2-(dimethylamino) ethyl)-methacrylamide (DMAEMA) (Figure 2) [29]. Due to the competitive binding between glucose and GEMA moiety with Con A, the microgels presented a fast response when glucose concentration was changed. In addition, small changes in the environmental pH regulate the insulin release profile: the insulin diffusion coefficient increases by raising the glucose concentration and decreasing the pH value (Figure 2). The ionization of amino groups at a low pH value determines an increase in the swelling degree of the polymer network (PDMAEMA). At a high pH value, the content of ionic DMAEMA units decreases and the free amino groups can be involved in hydrogen bonds, forming a compact network which limits the insulin release.

Glucose-sensitive phase-reversible hydrogels based on poly(hydroxyethyl methacrylate) (PHEMA) and Con A were prepared and used to control insulin and lysozyme release [115]. Due to sufficiently fast sol–gel transition occurrence, an “*on*/*off* ” mechanism was triggered; the glucose diffusion rate was the determining step, giving thus the possibility to regulate the insulin rate of release.

The copolymers offer an optimum balance between the multivalency of carbohydrate and the possible accessibility of lectin [116]. Glucose-sensitive hydrogels were obtained by mixing appropriate concentrations of Con A with poly(acrylamide-*co*-ally1 glucose) [95] or poly(vinylpyrrolidone-*co*-ally1 glucose) [117]. The glucose-sensing abilities of these hydrogels and the glucose-dependent gel–sol phase transition were evaluated and discussed. The key factors in the network or sol phase formation (which influence the sensitivity to glucose) were the concentrations of the copolymer and Con A, as well as the content of glucose bound to the copolymer chains. The non-covalent interactions between Con A and copolymer-bound glucose ensure the reversibility of the sol–gel transition. The gel–sol phase transition occurs if the concentration of free glucose is at least four times higher than those contained by the copolymer-attached glucose; this limit is sensitive to copolymer concentration, being influenced by the interaction of Con A with the copolymer chains. The gel structure can be restored again by dialysis against water when the free glucose is removed from the sol phase.

#### 3.1.2. Composites of Con A and Polysaccharide Derivatives

Due to the interaction of terminal and non-terminal sugars with highly specific receptors in lectin, mixtures of Con A with polysaccharides are immiscible and lead to precipitates [118]. By selecting the appropriate conditions (composition, pH, temperature, the characteristics of the gel membrane for solute diffusion, etc.), the reversible sol–gel transition occurs. The sol or gel formation is the result of the competitive displacement of glucose-bearing polysaccharide from Con A caused by the presence/removal of glucose, controlling in this way the diffusion of the drug existing in a reservoir [102]. The most frequently used polysaccharide structures for preparing glucose-responsive composites are dextran, chitosan, polysucrose, glycogen and pullulan, especially their derivatives and combinations with other macromolecules.

##### Dextran and its derivatives

Dextran (Dex) belongs to complex branched glucans with the main chain consisting of α-1,6 glucosidic bonds and random branches formed by α-1,2 or α-1,3, depending on the species of origin. It was used in a native or modified state in combination with Con A and other polysaccharides for preparing glucose-responsive materials.

Aqueous dispersions of Dex and concanavalin A undergo a sol–gel transition sensitive to changes in the glucose concentration with a fast response (less than 2 min). A biosensor was developed based on the electrical signal that occurs during the dissociation of Con A from Dex due to the competitive action of glucose, either by changing the lectin concentration by dissociation or by changing the viscosity of the liquid phase containing high concentrations of long Dex macromolecules and Con A [119].

Taylor et al. [120] successfully in vivo tested an implantable artificial pancreas of a biodegradable and biocompatible Dex/Con A complex on a live diabetic domestic pig, proving fast responsiveness, high resolution and the possibility of intramuscular/subcutaneous injection. Li et al. [121] designed a glucose biosensor based on the specific biorecognition of Con A, in combination with phenoxyl-dextran (DexP) and gold nanoparticles deposited on the electrochemically reduced graphene oxide (ERGO). Con A was covalently linked to gold nanoparticles and, when the glucose concentration is changed, competitive association/dissociation in the glucose/Con A/Dex system occurs. Due to the convenient signal transduction, this sensor presents a high potential for diabetes diagnosis.

Some glucose-sensitive microporous hydrogels were synthesized by the photopolymerization of glycidyl methacrylate modified dextran (DexG), modified concanavalin A (Con A–E) and poly(ethylene glycol) dimethacrylate (PEGDMA) [26]. In these networks, glucose sensitivity is controlled by the content of PEGDMA. The isothermal titration calorimetry data corroborated with the dynamic equilibrium theory of ligand binding were used to explain the binding interactions of the Con A/sugar-based systems and insulin delivery mechanism [26,81].

**Figure 3:**
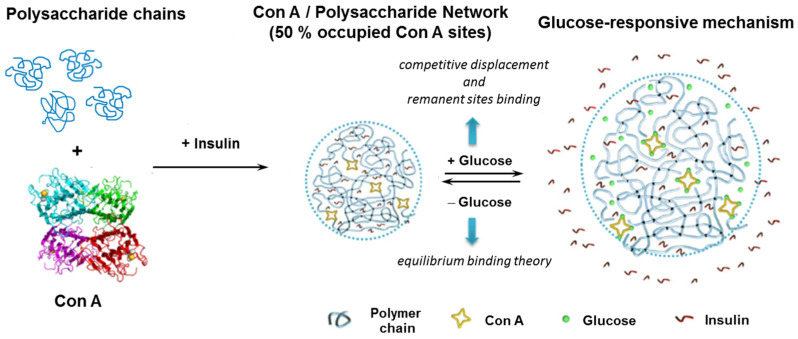
Glucose-responsive principle of Con A/polysaccharide system. Adapted with permission from [81], copyright 2019, Elsevier.

The competitive binding between polysaccharide chains and glucose with Con A is illustrated in Figure 3. The glucose-responsiveness of the Con A/sugar-based systems is influenced by the glucose concentration, the remaining sites of Con A and terminal groups on polysaccharide chains [81].

**Figure 4:**
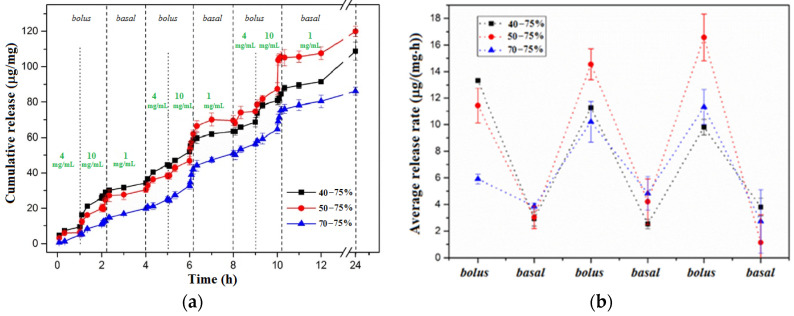
(**a**) Cumulative insulin release from Con A/DexG gel [μg/mg] for 24 h with three boluses simulating meals during day; (**b**) average rate of insulin release [μg/(mg·h)] as function of bolus and basal glucose concentration during cycle of 24 h. Adapted with permission from [81], copyright 2019, Elsevier.

Due to the steric hindrance within the network, in the Con A/DexG microgels, the four sugar interaction sites are not fully occupied. Therefore, when a high concentration of free glucose (for example 4 mg/mL) diffuses into the network, the competitive displacement of glucose from the Con A/DexG gel can occur due to the affinity of Con A for glucose, similar to the affinity of terminal groups of substituted dextran for Con A. On the other hand, the glucose molecules bind the Con A sites, thus the concentration of available Con A decreases and the Con A/DexG complex dissociates. If the concentration of free glucose decreases to a very low level (below 1 mg/mL, considered the limit of normal glucose level), an increase in the available Con A sites occurs. The terminal moieties of DexG can bind the available Con A sites and the Con A/DexG network reforms. The insulin release tests revealed that the microgels with 40, 50 and 70 occupied sites of Con A and a 75% percentage of reacted DexG molecules preserve bolus and insulin release as a function of glucose content. The released insulin remains active, maintaining the secondary and tertiary structure. The network composition influences the release rate and released insulin amount (Figure 4) [25,27,81].

Carboxymethyl-dextran (CM-DEX)-based hydrogels are versatile sensors interacting with proteins, nucleic acids or carbohydrates. The unique feature of CM-DEX hydrogels is an efficient electrostatic binding before covalent immobilization, a process which is sensitive even at low ligand concentrations. Aldehyde dextran sulfonate was also used to prepare biosensors. The aldehyde group is responsible for covalent bonding, and the negative sulfonate group provides electrostatic attraction with the polycations. The ratio between the aldehyde and sulfonate groups in the hydrogel matrix allows for the control of binding types (covalent, electrostatic or both) [121].

Other Con A/Dex systems were designed as interpenetrated networks including modified crosslinked poly(N-isopropylacrylamide) which was physically entangled with Con A and dextran sulfate. The network swelling is induced by anionic inclusion, and the shrinking is caused by uncharged pyranoside, attributed to the displacement of dextran sulfate from the Con A sites [122].

Hydrogel membranes were also constructed from crosslinked dextrans to which Con A was coupled via a spacer arm [66,97]. The addition or removal of glucose determines reversible changes in the porosity of this hydrogel, influencing the diffusion rate of the solute. A good permeability was depicted at 37 °C and pH = 7.4 [97].

##### Chitosan and its composites

Chitosan (CS) is a polycationic polysaccharide derived from chitin consisting of β-1,4-linked 2-amino-2-deoxy-D-glucose with a variable N-acetylation degree, that undergoes a protonation of free amino groups in an acid environment determining the electrostatic swelling of the macromolecular coils [123,124]. Chitosan is non-toxic, biocompatible and biodegradable with bacteriostatic, fungistatic, hemostatic and antiulcerous activity [123,125].

**Figure 5:**
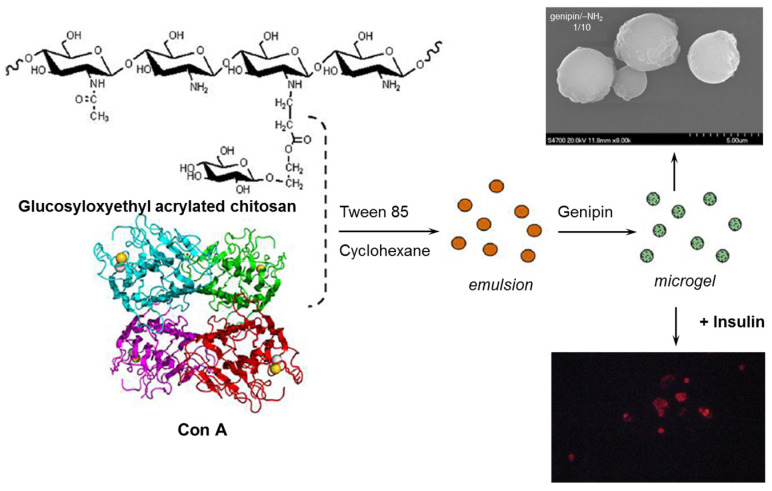
A schematic presentation of the fabrication of Con A/chitosan derivative microgels by reversed-phase emulsion. The morphology of microgels for a genipin/-NH_2_ ratio of 1/10 and a fluorescence microscopy image after insulin loading were included. Adapted with permission from [65], copyright 2014, Elsevier.

Microgels with different compositions of Con A and a chitosan derivative (glucosyloxyethyl acrylated chitosan) crosslinked with genipin were obtained by reversed-phase emulsion [65]. The denatured protein was reactivated by using Ca^2+^ and Mn^2+^ cations, in order to ensure Con A‘s interaction with the chitosan derivative. Spherical microgels with a diameter less than 5 μm were obtained for a genipin/-NH_2_ ratio of 1/10, being able to immobilize Con A and to restrict its leakage (Figure 5). The pH of the environment influenced insulin loading; the maximum encapsulation efficiency was registered at pH = 6.5. This system was tested for self-regulated insulin delivery. Con A forms preferentially a complex with free glucose, reducing the crosslinking points of the chitosan derivative. This is a reversible process influenced by glucose concentration.

By using ionic crosslinking with hydroxypropyl methylcellulose phthalate (HPMCP), chitosan nanoparticles were formulated as a pH-sensitive system, and it was evaluated for the oral delivery of insulin [126].

Glucose-responsive microparticles based on chitosan, Con A and Dex were prepared by coupling Con A with chitosan microparticles via a Schiff base reaction, and further, a Dex layer was incorporated via specific affinity [8]. These composite microparticles were used for self-regulated insulin delivery. Insulin was loaded through electrostatic and intermolecular interaction, and its release occurred as a response to changes in glucose concentration.

Insulin-loaded glucose-responsive microspheres, prepared by the high-speed shear emulsion crosslinking of DexG, PEGDMA and Con A, were integrated into chitosan hydrogels to produce a synthetic artificial pancreas [25,120,127]. In vitro insulin release from the hybrid composite scaffolds showed a prolonged and controlled delivery of drug as compared with the free microspheres, with regulated basal and bolus insulin release in response to glucose concentration levels.

A thermoresponsive composite hydrogel was designed by using chitosan, Pluronic F127 and Con A via 1-ethyl-3-(3-dimethylaminopropyl)-carbodiimide (EDC) and N-hydroxy succinimide (NHS) coupling (by the covalent linking of -NH_2_ from chitosan and Con A to the –COOH of carboxylated Pluronic F127) [34]. The prepared hydrogel revealed a sol–gel transition in physiological conditions and good injectability (shear-thinning behavior for a maximum force of 4.9 ± 8.3 N in a 26G needle). Also, this glucose-responsive hydrogel has shown sustainable insulin release during 7 days of about 97% via fluorescence spectrophotometry at 305 nm in simulated glycemic media (4 mg/mL and 10 mg/mL).

##### Glycogen and Polysucrose

Improved self-regulating drug delivery systems, using covalently modified glucose-sensitive gels based on Con A and a polysaccharide displacement mechanism, were reported. The insulin delivery characteristics of glycogen-based gels were examined by Tanna et al. [96]. Glycogen is a multibranched polysaccharide; its structure is based on α-1,4 glucosidic links with one 1,6 branching at approx. 10 glucose units. The number of branches influences the magnitude of the polysaccharide–Con A interactions, the gel structure being different as compared with that of Dex gels. Con A was covalently linked to glycogen by using the periodate method. Higher glucose sensitivity was observed for the glycogen-based hydrogel membranes as compared with Dex-based hydrogels. The insulin delivery was reversible, triggered by the increased content of glucose [96].

By isothermal titration calorimetry, Li and co-workers [128] have shown that the binding affinity of Con A/glucose in solution is considerably higher in the presence of polysaccharide. They demonstrated that the low affinity of glucose with Con A was unable to trigger the dissociation of the Con A/glycogen binding at the physiological pH value (pH = 7.4).

Polysucrose (α-D-glucopyranosyl-β-D-fructofuranoside) is a synthetic, water-soluble, non-toxic polymer, stable in alkaline and neutral solution resistant to intestinal enzymes. The molecules have a branched structure with a high content of hydroxyl groups giving a good solubility in aqueous solutions. A gel membrane based on polysucrose and Con A was used to design a self-regulating device for insulin delivery based on the displacement of the polysaccharide from the Con A receptors in the presence of glucose [102]. The viscosity decreases by incoming glucose and increases again by the elimination of glucose, controlling the diffusion rate of insulin. It was shown that for an aqueous insulin reservoir, the magnitude of the response can be controlled through the formulation parameters, temperature and path length of the membrane gel. The use of a non-aqueous reservoir (*n*-octanol) for the hydrophobic physical complex insulin–sodium dodecyl sulfate, improves the switch reversal and presents the advantages of higher reproducibility in the response and lower temperature sensitivity.

##### Pullulan and its derivatives

Pullulan is a linear glucan with the structure consisting of three glucose units connected by α-1,4 glycosidic-bonded maltotriose connected through an α-1,6 glycosidic linkage. This versatile water-soluble polysaccharide presents valuable properties (non-toxicity, biocompatibility, bioadhesion, biodegradability, etc. [129,130], being accepted by the Food and Drug Administration and European Union as a safe biopolymer.

The attractive interactions between pullulan and negatively charged macromolecules (polyanions [131] or proteins [132]), mediated by Na^+^ ions, determine the formation of a complex structure with implications in hydrogel stability and drug delivery in physiological conditions [133]. The oxidation applied to pullulan determines the conversion of the –OH groups into carboxylic ones and transforms the neutral macromolecules into valuable products with polyelectrolyte behavior, opening the route for a wide range of biomedical applications [134].

**Figure 6:**
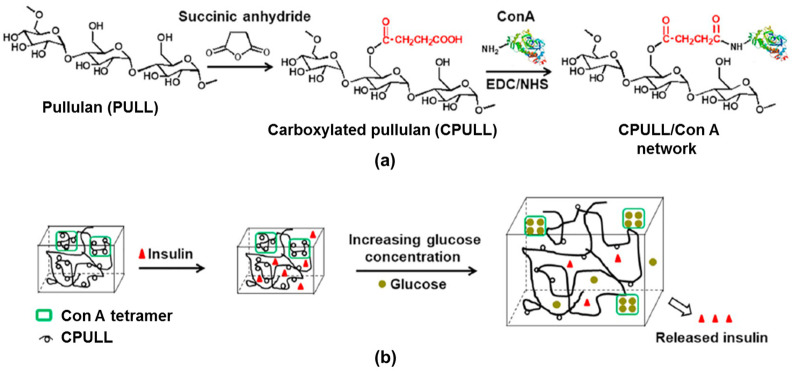
(**a**) The preparation of the carboxylated pullulan (CPULL)/Con A hydrogel. (**b**) The mechanism of the smart controlled release of insulin from the CPULL/Con A network. Adapted with permission from [24], copyright 2019, Elsevier.

**Figure 7:**
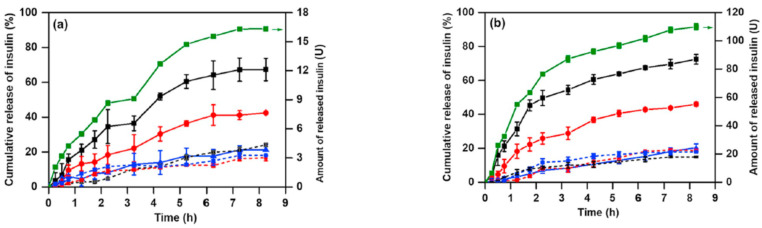
In vitro release profiles of insulin from the hydrogels in solutions with different glucose concentrations (at 37 ± 0.5 °C): (**a**) the insulin/CPULL/Con A hydrogel and the insulin/CPULL/Con A-N hydrogel with a loaded insulin concentration of 0.6 mg/mL; (**b**) the insulin/CPULL/Con A hydrogel and the insulin/CPULL/Con A-N hydrogel with a loaded insulin concentration of 10 mg/mL. Different release media were used: black curves: 20 mmol/L glucose in PBS (pH = 7.4) solution, red curves: 6 mmol/L glucose in PBS (pH = 7.4) solution, and blue curves: PBS (pH = 7.4) solution. Solid curves: the insulin/CPULL/Con A hydrogels. Dash curves: the insulin/CPULL/Con A-N hydrogels. Solid black, red and blue curves mean the percentage of cumulative release, and solid green curves mean the amount of released insulin. Adapted with permission from [24], copyright 2019, Elsevier.

The biospecific binding between Con A with –COOH groups of carboxylated pullulan (CPULL) avoids the leakage of Con A (Figure 6a) [24]. The CPULL/Con A bioconjugate was prepared by the EDC/NHS activation method and used to design a smart glucose-sensitive hydrogel. Con A dissolved in phosphate-buffered saline (PBS, pH = 7.4) was activated by adding 0.5 mM MgCl_2_, 0.5 mM CaCl_2_ and 0.5 mM MnCl_2_ (the method also used for chitosan [65] and dextran [66] derivatives). An inactivated hydrogel, denoted CPULL/Con A-N, used as a control, was prepared by a similar method from CPULL and Con A, without adding MgCl_2_, CaCl_2_ or MnCl_2_. The specific binding occurs only between the activated Con A residues and free glucose, determining a reversible gel–sol transition. The in situ insulin loading efficiency was 19 μg/mg for the inactivated insulin hydrogel samples and 122 μg/mg for the activated insulin hydrogels. When the glucose concentration increases, the hydrogel swells and releases insulin; when glucose levels become low, the hydrogel volume shrinkage decreases the insulin release rate. This behavior, triggered by the presence of glucose molecules, provides a switch for controlling the insulin diffusion rate (Figure 6b). Thus, due to the specific Con A/glucose binding, the hydrogel is able to release insulin in a hyperglycemic environment as a response to changes in glucose concentrations (Figure 7), being a promising material for improving diabetes therapy [24].

Recently, it was shown that the bioavailability of insulin in response to different BGLs can be considerably improved by using konjac glucomannan/Con A nanoparticles of about 500 nm, prepared by chemical crosslinking in the presence of trisodium trimetaphosphate. These nanoparticles were able to control the blood sugar levels for about 6 h [135].

### 3.2. A Brief Presentation of Con A-Based Biosensors for Glucose Detection

Many efforts were carried out for the development of reliable and highly sensitive devices able to monitor the glucose concentration in biological fluids. The most common methods are based on electrochemical, optical, fluorescence or colorimetric detection [11,21,22,24,25,136,137]. Another classification of biosensors was conducted according to the principle of glucose recognition, as presented in Section 1. Usually, the biosensors are able to monitor the glucose from the blood but also from other biofluids (tears, saliva, interstitial fluid, sweat or urine), all representing suitable routes [2]. The main requirements for new approaches and devices proposed for glycemic control are minimal invasiveness, predictable improvements in clinical outcomes and a positive impact on patient health. Thus, new and accessible biosensors for diabetes theranostics are continuously evaluated [136,138,139,140,141].

The first Con A-based biosensor was patented by Schultz [142], application filed by the US Department of Health and Human Services. Con A was immobilized within a hollow dialysis fiber and linked via a single optical fiber to a fluorescence detection apparatus. Dex was selected as competing ligand that was labeled with fluorescein isothiocyanate (FITC). Labeled Dex cannot pass in and out of the fiber, whereas small molecules, such as glucose, can pass. When glucose displaces its polysaccharide competitor from the binding site, the free Dex raises the fluorescence intensity inside the fiber (a large increase in fluorescence emission at 520 nm). Blood glucose was measured within the physiological range [143]. The response time was subsequently improved (5–7 min) for 50 to 400 mg/dL glucose [144] and, further, the sensor was slightly adapted to transdermal glucose monitoring [145].

In the last 10–15 years, researchers have taken up this topic and the number of studies on glucose-sensitive hydrogels containing Con A has started to increase. A glucose-sensitive sensor consisted of a small hollow fiber implanted in dermal skin tissue containing Cy7 labeled agarose-immobilized Con A and free Dex. This sensor was tested by fluorescence resonance energy transfer measurements on long-wave, near-infrared emission [146].

**Figure 8:**
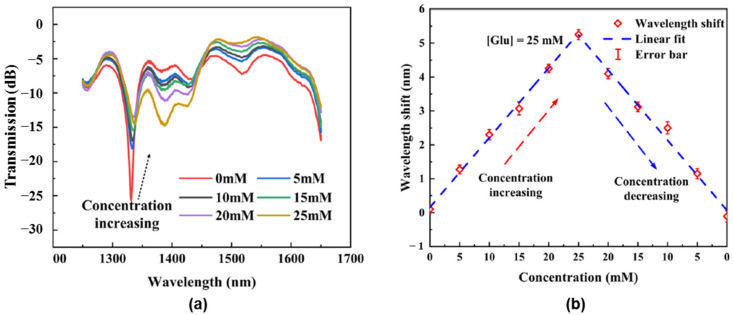
Test results of photonic biosensor for glucose detection: (**a**) spectral responses for glucose solutions with concentrations in range 0–25 mM; (**b**) wavelength shifts with increasing or decreasing glucose concentration. Adapted with permission from [138], copyright 2023, Elsevier.

A UV-curable hydrogel with glucose recognition was designed using the 3D printing technique. The photonic biosensor was composed of Con A, glycidyl methacrylate-modified dextran (DexG) and polyethylene glycol dimethacrylate (PEGDMA) and was proposed as a suitable device for detecting glucose and real-time continuous glucose monitoring in diabetic patients [138]. Due to the competitive binding of glucose molecules and DexG with Con A, the network undergoes a reversible swelling/deswelling in glucose solution, inducing changes in the refractive index and wavelength shifts in the transmission spectrum which are dependent on glucose concentration (Figure 8). The response time was 10–12 min with a sensitivity of 0.206 nm/mM, and the response range was up to a glucose concentration of 25 mM.

**Figure 9:**
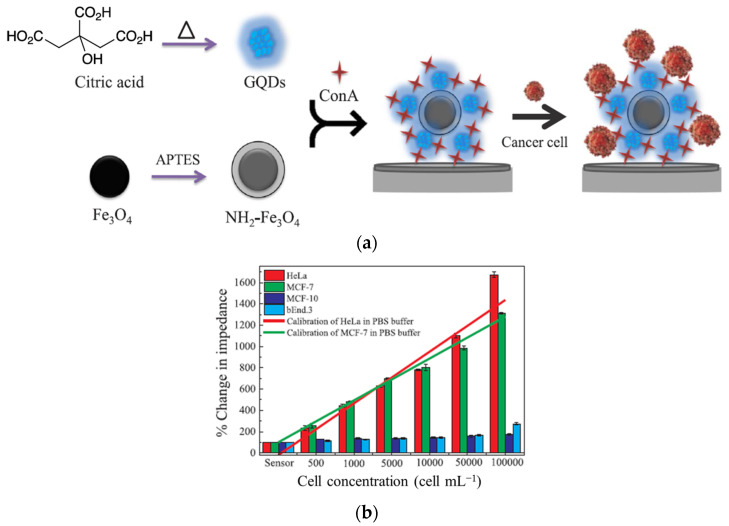
(**a**) The preparation of Con A/GQD@Fe_3_O_4_ for cancer detection; (**b**) the change in impedance and the linear relationship as a function of cell concentration in PBS buffer. Adapted with permission from [149], copyright 2018, Elsevier.

Selective and sensitive bioactive tools are required for improving glucose detection. Various polymeric glyconanoparticles with different functional groups and morphologies were prepared as promising biosensors. Among them, glyco-quantum dots (GQDs) [147,148] and gold nanoparticles (AuNPs) [149] were designed for in vivo or in vitro biosensing devices delivering optical and electrochemical signals. Chowdhury et al. [149] have developed a biosensor for the quantitative determination of cancer cells having high glycoprotein expression (Figure 9). Con A/GQD@Fe_3_O_4_ nanocomposites with specific selectivity in detecting HeLa and MCF-7 cancerous cells were prepared by anchoring covalently GQDs on the Fe_3_O_4_ surface followed by physically bonding Con A onto GQDs (Figure 9a). A linear correlation between the impedance and cancerous cell concentration (up to 246 cells/mL for HeLa and 367 cells/mL for MCF-7) in PBS buffer was found (Figure 9b). This allows for the quantitative detection of circulating tumor cells (CTCs) and an early diagnosis of cancer cells with high glycoprotein expression.

D-glucose immobilized by multi-wall carbon nanotube–polyaniline nanocomposites through the Schiff base reaction showed high binding sensitivity and excellent selectivity to Con A for concentrations in the range of 3.3 pM to 9.3 nM, with a detection limit of 1.0 pM [150]. The performances of this biosensor (stability and sensitivity) are due to the multiple sites of Con A with a high affinity for D-glucose.

**Figure 10:**
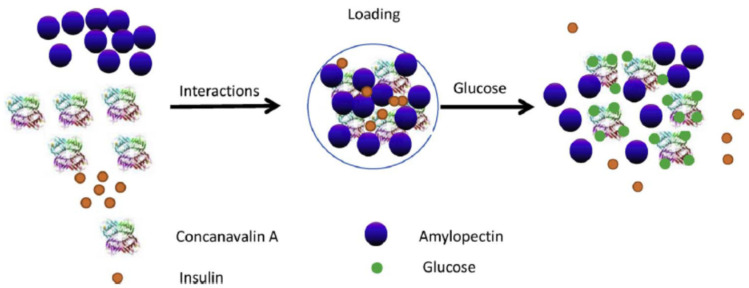
Schematic presentation of mechanism of insulin loading and release from nanoparticles based on Con A and amylopectin. Adapted with permission from [90], copyright 2017, Elsevier.

**Figure 11:**
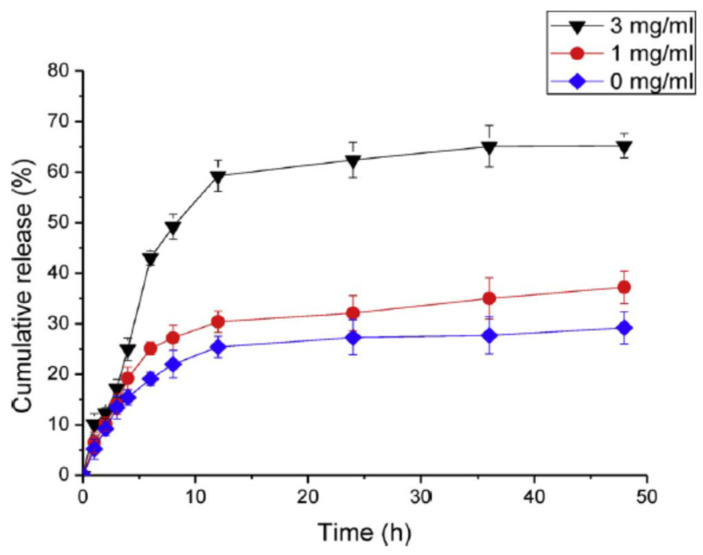
In vitro cumulative release of insulin in phosphate buffer solution (pH = 7.4) from Con A/amylopectin nanoparticles in 0, 1 and 3 mg/mL of glucose medium. Adapted with permission from [90], copyright 2017, Elsevier.

Con A has a higher affinity for glucose as compared with the glycosylated moieties of amylopectin. Nanoparticles based on Con A and amylopectin co-assembling were prepared by Chang et al. [90], and the effect of the Con A/amylopectin composition, pH and ionic strength on the behavior of the glucose-responsive system was investigated. The affinity was stronger for the Con A/amylopectin mass ratio of 3/1, pH = 5.2 and NaCl concentration of 0.12 g/mL. The possible mechanism of the insulin release of Con A/amylopectin is shown in Figure 10. Insulin was in situ loaded into Con A/amylopectin nanoparticles. The addition of glucose determines a competition with amylopectin from the nanoparticles to bind Con A, producing a break of the nanostructure and thus the insulin releases. The amount of glucose which is combined with Con A, and respectively the quantity of released insulin, increases in time during 10 h (Figure 11).

**Figure 12:**
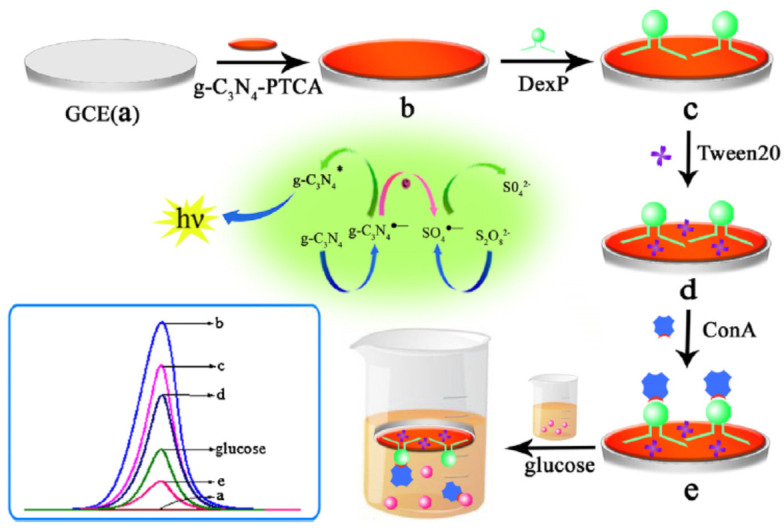
Schematic presentation of preparation steps of ECL sensor for glucose detection. With permission from [151], copyright 2015, Elsevier.

An electrochemiluminescence (ECL) sensor for glucose detection was prepared by taking into account the competition reaction between glucose and phenoxy dextran (DexP) for Con A binding sites [151]. DexP was immobilized by π-π interactions onto a glass carbon electrode (GCE) provided with 3,4,9,10-perylenetetracarboxylic acid (g-C_3_N_4_-PTCA) as a signal probe. Con A was bound onto the electrode through the specific interactions between DexP and Con A. By immersing this sensor into the glucose solution, glucose would compete with DexP for Con A. As the glucose concentration increases, a corresponding Con A amount leaves the electrode and gives a corresponding ECL signal, allowing for a sensitive determination of glucose (Figure 12) [151].

Another electrochemical sensor was constructed from a chitosan-functionalized graphene oxide (CS/GO) composite as a substrate and horseradish peroxidase immobilized on the CS/GO surface (via a Schiff base reaction) as an amplification reagent. Con A was specifically captured by D–mannose (D-man) (via a Schiff base reaction), forming a sandwich configuration. This electrochemical sensor with enzyme catalytic amplification used hydroquinone as an electrical mediator and was proposed for the sensitive detection of Con A with a detection limit of 1.24 × 10^−9^ mol/L [152].

**Figure 13:**
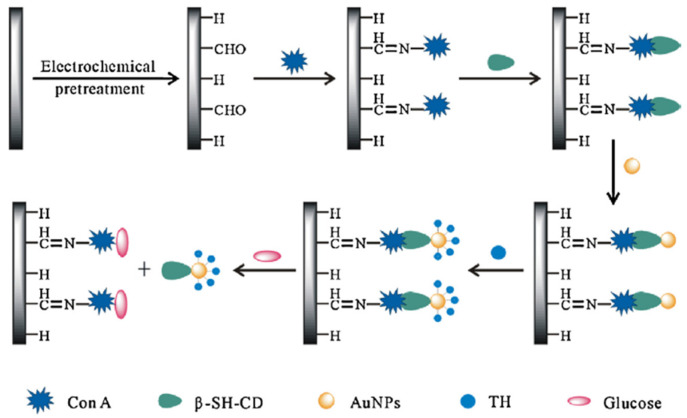
Schematic illustration of β-SH-CDs/Con A/AuNPs glucose biosensor. Adapted with permission from [153], copyright 2013, Elsevier.

**Figure 14:**
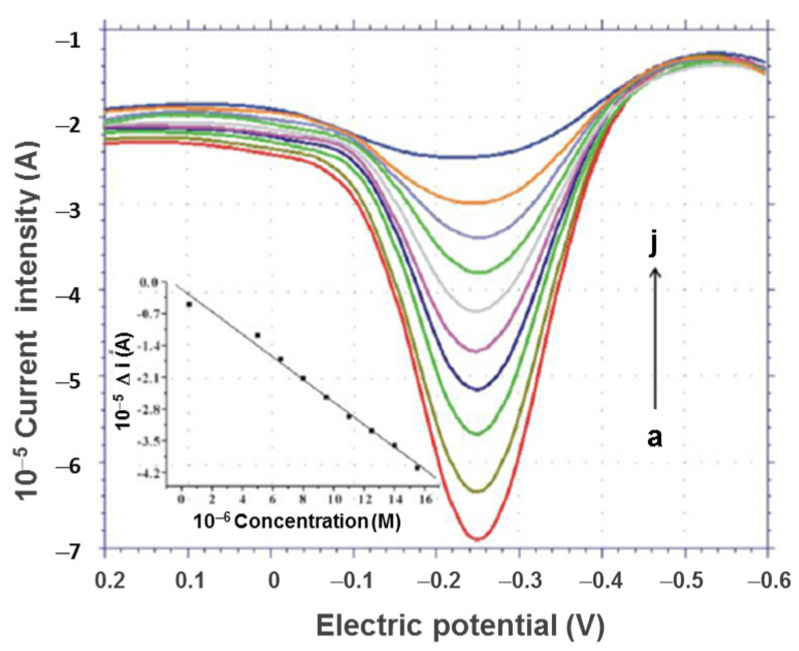
The differential pulse voltametric curves registered for the β-SH-CDs/Con A/AuNPs sensor at different D-glucose concentrations in 0.1 M PBS at pH = 7: (a) 0; (b) 5.0 × 10^−7^ M; (c) 5 × 10^−6^ M; (d) 6.5 × 10^−6^ M; (e) 8 × 10^−6^ M; (f) 9.5 × 10^−6^ M; (g) 1.1 × 10^−5^ M; (h) 1.25 × 10^−5^ M; (i) 1.4 × 10^−5^ M; and (j) 1.55 × 10^−5^ M. The inset shows the calibration plot of the difference in the peak current intensity versus glucose concentration. Adapted with permission from [153], copyright 2013, Elsevier.

The non-enzymatic detection of glucose was conducted by using a selective sensor based on thiolated β-cyclodextrins (β-SH-CDs) coupled with gold nanoparticles (AuNPs) for signal amplification (Figure 13) [153]. Con A with much stronger binding capacity towards D-glucose compared with β-cyclodextrins determines a decrease in the voltammetric signal of thionine (TH). Each oxidation wave for TH moiety was accompanied by a well-defined peak, and the registered current decreased with increasing the concentration of D-glucose from 5.0 × 10^−7^ M to 1.55 × 10^−5^ M (Figure 14) [153].

The polymeric nanoassemblies are promising routes in the design of different bioactive sensors for bio- and nanotechnologies, gene therapy and controlled drug delivery [47]. Porphyrin-based drugs are promising systems in photodynamic therapy, but they are generally poorly water-soluble and not suitable for intravenous administration. The delivery efficacy may be improved by conjugation with hydrophilic polymers, such as glycopolymers. Porphyrin/glycopolymer conjugates form micelles in the water (with a hydrophobic porphyrin as core and corona formed by two hydrophilic glycopolymers at the ends) with the great and specific binding ability of Con A [154]. The binding efficiency of these micelles is influenced by their size, as a result of the hydrophobic/hydrophilic balance and macromolecular architecture. The highest binding affinity per mannose functionality was achieved for stiff polymers, trying to match the hydrodynamic diameter of the polymeric structure to the binding sites of Con A [79].

Mannose-conjugated magnetic NPs presented high recognition ability toward Con A [155]. The polymeric glyco-nanoparticles showed an anticancer effect for cancer cells (K562), being suitable as theranostic agents for cancer imaging and therapy [80,148]. The anticancer effects become higher and more specific by binding Con A to the glyco-micelles, which are able to destroy the cancer cells under light irradiation [154,156] Another sensitive electrochemical glucose biosensor was developed by Ye et al. [157]. C60-fullerene was functionalized with tetraoctylammonium bromide (C_60_-TOAB^+^) deposited on the surface of a glassy carbon electrode (GCE). Con A was then linked to the electrode surface. The C_60_-TOAB^+^ composite film is able to undergo a reversible redox reaction, allowing for the determination of the salivary glucose level [157]. A disulfide-carrying polymer with pendent glucose residues, poly(2-methacryloyloxyethyl D-glucopyranoside), was placed on a colloidal Au-immobilized glass substrate and used as a sensing element of Con A, and its detection limit was 1.9 nM [158].

Multilayer films were prepared through a pH-sensitive self-assembling mechanism by using chitosan (CS) and Con A as the inner layer and poly (N,N–diethyl acrylamide) (PDEA) hydrogel as the outer layer that contains glucose oxidase (GOD) and horseradish peroxidase (HRP) enzymes. The resulting {CS/Con A}_n_-(PDEA-GOD-HRP) multilayer film with a binary structure was fixed on the electrode surface [159], this system being able to control the bio-electro-catalysis of glucose in the presence of enzymes.

The stimuli-responsive multilayer films were prepared by the layer-by-layer deposition technique using glycogen and Con A [100]. These films can be fully disintegrated in the presence of D-glucose, D-mannose and their derivatives, in aqueous solutions at neutral pH. This is due to the expulsion of glycogen from the binding sites of Con A as a consequence of the competitive binding of the free sugars to the binding sites of Con A in the film. Thus, glycogen and Con A films can be used as sugar-sensitive or sugar-sensitive delivery systems.

Another type of glucose-responsive insulin release sensor based on Con A was constructed by conjugating insulin to one or more glucose molecules, which can then tether insulin to the Con A constituted hydrogels [160]. The first research was reported by Brownlee and Cerami [108] who synthetized a maltose/insulin conjugate. A stepwise increase in glucose concentrations determined the increase in the intensity of the insulin release. Based on this concept, insulin was conjugated to other glucose-like molecules: maltose, maltotriose, mannotriose or mannotetrose [109].

Con A is one of the most widely used lectins with a wide applicability. The incorporation of Con A into glucose-responsive systems or biosensors is of current interest. Some relevant Con A-based systems are summarized in Table 1. In addition, immobilized Con A was used in the affinity chromatography purification of glycoproteins or cellular structures [67,72,161]. The specific interactions between Con A and carbohydrates represent the molecular basis of a series of biological recognition phenomena [161,162,163]. The involved mechanisms are useful for drug delivery applications as a response to chemical and biochemical stimuli, including pH changes, a variation in specific ion concentration and biomolecules’ recognition [164].

An intravascular electrochemical biosensor has been recently reported, suitable for in vitro and ex vivo glucose monitoring under homeostatic conditions, and this device, tested in simple buffers and human physiological fluids, is promising for preserving the normal glycemic level [165].

Oral or intramuscular/intravenous administration is the main approach for drug delivery in the clinical medical field but presents some shortcomings in terms of diabetes treatment. The oral administration of drugs involves the enzymatic degradation of the medicinal substance in the gastrointestinal system or in the liver [166]. However, intravenous administration would affect the daily lifestyle of diabetic patients, being dependent on hospital care. Transdermal drug delivery systems (TDDSs) use the microneedle (MN) method to penetrate the stratum corneum (SC), the outer layer of the skin. SC perforation leads to the formation of microchannels into the skin, without disturbing the nerves or blood vessels. Several studies have demonstrated that by using MNs, chemotherapeutic drugs, proteins or insulin can be delivered transdermally. MNs represent pain-free approaches to drug administration, being sensitive to stimuli and releasing drugs under controlled conditions (self-administration) [32,167].

Smart MNs are typically based on polymer matrices and comprise a wide range of compounds that are able to respond to environmental stimuli, such as the pH, redox potential, glucose and enzyme levels, temperature, electric field, light and mechanical stress. Due to the interception of internal or external stimuli, these systems respond by degrading, swelling, dissociating or cleaving the matrix, allowing for drug release. Materials used to obtain MNs require mechanical and biocompatibility specifications. Poly(vinyl alcohol) and poly(vinyl pyrrolidone) are the most used synthetic polymers for the MNs’ synthesis. Natural (macro)molecules are also used in order to prepare MNs due to their biocompatibility and ability to reduce the immune response of the MNs’ therapy. Among the natural molecules used are polysaccharides (hyaluronic acid, chitosan, pullulan, sodium alginate), proteins (gelatin, silk-fibroin), amino acids (lysine) and organic acids (folic acid, tartaric acid, lactic acid, polyglycolic acid) [10]. When the MNs come into contact with the dermal microcirculation, the metabolic changes in the physiological environment can be detected. In the case of patients with diabetes, the blood glucose level must be constantly monitored so that the antidiabetic drugs can be administered on time.

Despite the fact that there is no maximum dose for insulin, studies have demonstrated that using the recommended amounts of insulin can help achieve glycemic targets [168]. A suitable management of type 2 diabetes follows the recommendation of the American Diabetes Association (ADA) and the European Association for the Study of Diabetes (EASD) to stabilize the glycemic levels in the normal range [169,170,171].

According to the EASD and ADA standards of care, an individualized plan is required for the initiation and modification of insulin therapy.

## 4. Conclusions and Future Perspectives

Regular insulin injections cannot prevent the complications of diabetes, so the development of insulin delivery devices is an ongoing concern of researchers. Various studies have been conducted on polymer hydrogels as possible devices to control insulin release. A suitable insulin delivery system should be able to monitor blood glucose levels (BGLs) and release the required amount of insulin.

Concanavalin A (Con A) is a glucose-binding protein, extracted from plant lectins, with a tetrameric structure which can reversibly be attached to glucose and other saccharides, acting as a crosslinker. Con A can be integrated into a network structure, forming a glucose-binding element that can control the release of insulin when glucose concentration increases. Variations in the swelling degree influenced by glucose concentration facilitate controlled insulin release. Thus, Con A represents an attractive receptor for the next generation of non-enzymatic sensors [106].

Many efforts were undertaken to produce chemically and mechanically stable membranes/sensors capable of specific permeability changes in response to glucose concentration changes determining the controlled release of insulin. The present paper discusses the most important approaches used for the development of Con A-based glucose-sensitive hydrogels with self-controlled drug delivery, able to adjust the delivery of insulin in response to changes in glucose levels. An important requirement is to obtain a negligible Con A leakage over long periods of time (the leakage is more significant in a low viscosity phase). To avoid this leakage, Con A is covalently bonded to a polymer. Another specific requirement of hydrogel synthesis was to maintain a pH value below 9 during the reaction, in order to minimize Con A inactivation during coupling.

The major drawback for the use of Con A-based hydrogels in biomedical application is the irrelevant cytotoxic effect [21,94]. The recent therapeutic options based on the theranostic principle for diverse types of diabetes and cancer, as alternatives to traditional methods, enhanced the patient’s survival rate. However, patients with diabetes still suffer due to different constraints. If the blood glucose level is not well controlled and maintained in the normal limits, below 100–125 mg/dL (5.6 mmol–6.9 mmol/L) in venous plasma, serious complications appear, such as diabetic foot ulcers [1,172], heart disease, kidney failure, cerebral infarction, atherosclerosis or blindness [2,173,174]. In order to minimize these complications, patients must monitor and self-regulate their blood glucose concentration by the continuous release of the required insulin amount. The research is now oriented to controlling insulin release and tailoring it for each patient in personalized diabetes care approaches [25,81,175,176,177,178,179].

Nature lessons can be applied to develop functional hydrogels by the physical and chemical modification of natural polymers and proteins, in particular Con A which is able to bind carbohydrates in a specific and reversible manner [180], or the development of environmentally friendly polymer networks [9]. The personalized treatment by using targeted pharmaceuticals is one of the quickly emerging strategies for overcoming problems in non-invasive glucose monitoring [2,136,137,139] and insulin delivery [3,4,6,8,135], particularly for high levels of glucose in the body.

One of the goals of chemists and pharmacists would be a deep search for the construction of multivalent Con A/polysaccharide sensors with precise geometries and minimal invasiveness which must be highly effective in binding free glucose in the body. Comprehensive information concerning the most promising systems, such as the biocompatibility and biostability/biodegradability in physiological conditions, should be provided. Nevertheless, it must be admitted that the design, manufacture, characterization and optimization of suitable formulations in accordance with in vitro and in vivo tests require a huge effort and time [3,180,181,182,183].

The approaches covered in this review refer mainly to polymer/Con A hydrogels that are excellent candidates for insulin delivery because of unique characteristics:

- A fast and sudden response of the system to changes in glucose concentration;
- The drug can be dispersed uniformly into the hybrid network;
- The macromolecular chain dynamics (i.e., the rate of entangled or disentangled structure formation) favor the reversible interactions with glucose and Con A and, consequently, the insulin delivery;
- Suitable stiffness can be achieved, with tunable rheological and mechanical properties.

However, even if such complex systems are deeply investigated by a series of groups, the majority of glucose biosensors are far away from a possible implementation in diabetes management. Some problems may arise during in vivo tests under physiological conditions and decrease or compromise hydrogel performances [9,165,178,181,182,183]:

- Tests of glucose responsiveness after applying as many cycles as possible (hundreds or thousands of cycles) and a careful analysis of the reproducibility of results;
- Tests of hydrogels’ biocompatibility and biodegradability;
- An appropriate amount of released insulin for various concentration gradients; the long-term administration of higher insulin doses produces unwanted hypoglycemic effects;
- Adequate oxygen diffusion through the hydrogel matrices and biological fluids;
- Reduction in the interferences of physiologically relevant electroactive species (such as aspartic acid, uric acid) and active substances included in glucose biosensors;
- Avoid the non-specific interactions (such protein adsorption) on the biosensor surface that could lead to the biofouling or passivation of the surface.

In conclusion, an ideal in vivo glucose biosensor must be non-invasive and biocompatible, with high specificity, short response time, stability under storage and using conditions, low price and suitable for mass production. Despite the fact that the price for the high-purity Con A sample is high, a glucose sensor requires a small protein amount. This lectin provides a biologically derived method for glucose-sensing hydrogels that can be further included in a biosensor with a high ability to reversibly bind glucose molecules [9,44,86,87]. The small number of studies on Con A as a glucose biosensor has been attributed to the limited data on the toxicity of this protein and the required chemical modification of insulin [184]. A more extensive use of Con A for biosensors production is desirable, as an alternative for improving diabetes management.

## Figures and Tables

**Table 1:** Con A-based glucose-responsive materials.

	Complex	Administration	References
	Dex/Con A	Intramuscular injection	[120]
	DexP/Con A/AuNP/ERGO	Skin sensor (diagnostics)	[121,151]
	DexG/Con A–E/PEGDMA	Skin sensor (insulin carrier)	[26]
*Glucose-responsive*	Glucosyloxyethyl acrylated chitosan/Con A	Self-regulated insulin delivery	[65]
*materials*	DexG/PEGDMA/Con A/Chitosan	In vitro insulin delivery	[25,127]
	Chitosan/Pluronic F127/Con A	Injectable (controlled release)	[34]
	Polysucrose/Con A	Self-regulating membrane	[102]
	CPULL/Con A	Injectable (controlled release)	[24]
	Glycidyl methacrylate modified Dex/Con A	Oral dosage forms	[26,81]
